# Josh Billings Says

**Published:** 1873-04

**Authors:** 


### JOSH BILLINGS SAYS

To young gentlemen: "Keep cool. Cultivate your mustache. Be polite to your rich aunt, if you have one. Study

Hall's Guide tew Health,

And shun all grass-widders.

